# Fifty Years of Handedness Research: A Neurological and Methodological Update

**DOI:** 10.3390/brainsci14050418

**Published:** 2024-04-24

**Authors:** Anna Rita Giovagnoli, Alessandra Parisi

**Affiliations:** Fondazione IRCCS Istituto Neurologico Carlo Besta, Via Celoria 11, 20133 Milano, Italy; alessandra.parisi@istituto-besta.it

**Keywords:** handedness, hand, foot and eye laterality, immune-mediated diseases, Edinburg Handedness Inventory, Italian normative data

## Abstract

Handedness, a complex human aspect that reflects the functional lateralization of the hemispheres, also interacts with the immune system. This study aimed to expand the knowledge of the lateralization of hand, foot, and eye activities in patients with immune-mediated (IM) or other (noIM) neurological diseases and to clarify the properties of the Edinburgh Handedness Inventory (EHI) in an Italian population. Three hundred thirty-four patients with IM or noIM diseases affecting the brain or spine and peripheral nervous system were interviewed about stressful events preceding the disease, subjective handedness, and familiarity for left-handedness or ambidexterity. The patients and 40 healthy subjects underwent EHI examination. In the whole group of participants, 24 items of the EHI were classified into five factors (Hand Transitive, Hand Refined, Hand Median, Foot, Eye), demonstrating good reliability and validity. Chronological age had a significant influence on hand and foot EHI factors and the laterality quotient (LQ), particularly on writing and painting. In the patient groups, EHI factors and the LQ were also predicted by age of disease onset, duration of disease, and family history of left-handedness or ambidexterity. No differences were found between patients and healthy subjects, but pencil use scored significantly lower in patients with IM diseases than in those with noIM brain diseases. These results demonstrate that the lateralization of hand and foot activities is not a fixed human aspect, but that it can change throughout life, especially for abstract and symbolic activities. Chronic neurological diseases can cause changes in handedness. This may explain why, unlike systemic immunological diseases, IM neurological diseases are not closely associated with left-handedness. In these patients, the long version of the EHI is appropriate for determining the lateralization of body activities to contextualize the neurological picture; therefore, these findings extend the Italian normative data sets.

## 1. Introduction

Handedness is the preference for using one hand for unimanual tasks or demonstrating greater efficiency in performing such tasks with one hand, and a recent meta-analysis showed that 89.4 percent of the general population is right-handed [1]. This reflects hemispheric lateralization, that is, functional and structural asymmetries in the brain for basic and complex behaviors. Each hemisphere is dominant in particular functions over the other [2], but this does not strictly correspond to handedness, as a small percentage of left-handed individuals show the type of hemispheric lateralization (e.g., language) observed in fully right-handed individuals [3,4,5,6].

Handedness expresses asymmetry of movement control and underlying neural systems [7,8,9] corresponding to differences in brain morphology [10]. Jang et al. [11] observed that the right putamen and left globus pallidus of non-right-handed people were significantly larger than those of right-handed people and that the degree of hand laterality had a negative correlation with the volume of these nuclei. Notably, the basal ganglia of non-right-handed people were larger than those of right-handed people, suggesting that left-handed people have better motor control than right-handed people. Indeed, the putamen and globus pallidus play an important role in motor control [12] and cognitive control [13]. The putamen is involved in motor performance, movement sequences, and motor preparation because of its connections with cortical structures involved in the control of body movements [14,15,16,17]. The globus pallidus is involved in the constant regulation of voluntary movements such as speaking and walking fluently [18,19]. In patients with Alzheimer’s disease (AD), cognitive decline has also been associated with loss of putamen volume [20], which is why Jang et al. [11] hypothesized that left-handed people, having greater putamen volumes than right-handed people, may be more resistant to cognitive decline in AD. In addition, nonheritable changes have been found in left-handed individuals in the asymmetry of the mean thickness of the postcentral cortical gyrus and inferior occipital cortex, which are associated with the sensorimotor and visual functions of the hand. Surface asymmetries in language-related regions that are heritable may be related to both hand preference and language development, whereas nonheritable asymmetries in the sensorimotor cortex may result from hand preference [21]. There are also differences in intrahemispheric and interhemispheric white matter structural connectivity between left- and right-handed people. Handedness has been linked to asymmetries in certain frontoparietal association pathways, which are crucial for visuomotor and visuospatial processing, rather than the corticospinal tract, which is responsible for motor execution [22]. In addition, Sha et al. [21] observed that, compared with right-handed people, left-handed people show less leftward and more rightward shift in the thickness asymmetry of some cortical areas (fusiform gyrus, anterior insula, middle anterior cingulate, and precentral, postcentral, and inferior occipital cortex). These findings suggest a general tendency for a shift in neural resources toward the right hemisphere, which is often responsible for dominant hand control in left-handers. The authors also identified a link between hand dominance and asymmetrical areas of the brain surface in key regions responsible for language processing, particularly the anterior insular cortex/pars triangularis. These regions are known to be activated in the left side during sentence-level language tasks. The anterior insula plays a role in determining language dominance during early development by integrating social, emotional, and attentional systems during language learning. A polygenic predisposition to left-handedness has been also linked to certain asymmetries in the fusiform cortex and anterior insula. In fact, increased genetic predisposition to left-handedness is associated with decreased leftward asymmetry or increased rightward asymmetry in these areas.

The development of handedness depends of many interacting factors, such as genetics, cultural influences, parental education, and imitation behavior [23,24,25,26]. A large study has identified 48 genetic variants associated with handedness [27]. Genetic studies have also suggested that environment exerts an influence on handedness development [28], while behavioral studies have considered a genetic influence [23,24]. The minor lateralization in left-handers compared to right-handers, in terms of brain activation and behavior, can be explained by the pressure exerted by the environment to induce right-handedness [23,29,30,31,32]. In addition, some gestures and actions require the use of the right hand even in left-handers, leading to practice with the non-dominant right hand. The greater symmetry in movements resulting from the conflict between the intrinsic dynamics and the demands of the environment could also explain the greater symmetry in the brain activation and anatomy of left-handers [31].

The immune system is self-regulated and affected by psychophysical stressors [33,34] and environmental stimuli [35]. The brain mainly influences the immune system through the autonomic nervous system and the neuroendocrine system [36]. The sympathetic and parasympathetic structures directly innervate lymphoid tissues such as the thymus, spleen, lymph nodes, and mesenteric patches [37], and various neuroendocrine factors modulate the immune functions [38,39]. This communication is bidirectional, as immune modulators, especially cytokines, influence the autonomic and neuroendocrine systems [40]. In this framework, the question arises whether the central nervous system also asymmetrically modulates the immune responses. In a study on in vivo magnetic resonance imaging by Cerqueira et al. [41], it was observed that chronic treatment with glucocorticoids led to volumetric reductions in the left cingulate cortex, indicating the higher susceptibility of the left mPFC to the impact of elevated corticosteroid levels and, potentially, stress. This heightened vulnerability of the left hemisphere to glucocorticoid effects has also been documented in the human brain, with a study by MacLullich et al. [42] linking dysregulated HPA axis activity (hyperactivity) to a smaller volume of the left cingulate, as opposed to the right hemisphere.

Animal studies have demonstrated opposite immunological responses after unilateral brain stimulation or damage [43,44,45,46,47]. Decreases in immunological parameters, such as natural killer cell activity, T-lymphocyte proliferation, and immunoglobulin G antibody production, were shown after left hemisphere damage, while no immunological change or even improvement in immunological parameters were found after right hemisphere damage [43,44,45,48]. The functional asymmetry of the immune system and the role played by the brain hemispheres in the development of humoral immune responses have also been demonstrated in F1 mice [49].

Research in humans stems from Geschwind and Behan’s theory based on the association between immune-mediated (IM) disorders and left-handedness in left-handed population surveys and studies of patients with IM disorders [50]. These authors hypothesized that the normal development of the brain hemispheres may be modified by high prenatal testosterone levels. This may promote the growth of right hemisphere regions and slow down homologous left hemisphere regions [51,52,53], thus stimulating an abnormal dominance pattern (non-right-handedness and the atypical dominance of language and visuospatial abilities). Such an abnormal hemispheric dominance would predispose one to immunological diseases, as suggested by the high incidence of left-handedness among individuals with developmental and IM disorders [50,54].

Notably, left-handedness is not an index of abnormal brain dominance, and other factors, such as sex and humoral regulators, can influence immune function [55]. Women are more likely than men to be affected by IM disorders such as systemic lupus erythematosus (SLE), multiple sclerosis (MS), myasthenia gravis, thyroid disease, arthritis, and topical allergies [56]. These findings and the involvement of female sex hormones in the pathogenesis of autoimmune diseases [57] refute the role of testosterone in immune disorders and brain asymmetry.

As for the regulators of immunity, Lengen et al. [58] examined markers of cellular immunity, observing a significant reduction in inflammatory CD3+ T cells (total T cells) and CD4+ T cells (T-helper cells), HLA-Dr (major histocompatibility complex, MHC-II, antigen-presenting cells), and CD19+ (B cells) and CD16/CD57+ (natural killer cells) cells in left-handed people compared to right-handed people. Moreover, the number of CD3+ T cells predicted left-handedness. Given that MHC stimulates no-self cells to trigger an immune response and T lymphocytes recognize no-self triggering immune responses, these findings highlight the link between handedness and autoimmune responses. In addition, the hemispheric side was related to postoperative changes in T-cell indices in patients undergoing epilepsy surgery: lymphocyte counts; total T lymphocytes, helper T lymphocytes, cytotoxic/suppressor lymphocytes; and total suppressor lymphocytes decreased after resections in the language-dominant hemisphere but increased after nondominant hemisphere surgery [59]. Dopamine is an important neuro-immune regulator that counteracts T-cell function: it can modulate T-cell proliferation, INF-c secretion, and matrix metalloproteinase-9 mRNA production in MS patients [60]. Other neuro-immune regulators (catecholamine, serotonin, noradrenaline) have been linked to autoimmune pathogenesis in SLE, MS, and rheumatoid arthritis [61,62,63,64,65].

The Edinburgh Handedness Inventory (EHI) [66] is the most widely used measure of hand, foot and eye laterality. It includes ten goal-ended hand movements and two movements performed with the eye and foot. As written by Oldfield, “Doubtless the inventory is not ideal, but it is simple and provides one quantitative measure of handedness backed by a known distribution of values in a reasonable sized normal population. And it gives some insights into the inter-relationship of individual items of the kind in such devices”. Early studies suggested that the gestures included in the EHI load on a single factor (handedness factor), with the exception of opening a box and manipulating a broom [67,68], and recent studies [69,70] have confirmed the one-factor solution. The EHI measure was also found to be sensitive to sociodemographic and cultural factors, but few studies have compared participants’ self-reported EHI scores with performance measures [71,72,73]. Ruck and Schoenemann [74] showed, in particular, a poor match between EHI scores and Rolyan’s nine-hole board and grip strength scores in 1179 healthy subjects. The EHI is also used in neuroimaging [75] and clinical neurology to evaluate the association between handedness and sleep disorders [76], migraine [77], epilepsy [78], and AD [79]. Other studies have used the EHI to describe participants or to contextualize the cognitive profile in neurological [80,81], neurosurgical [82,83], and psychopathological conditions [84].

In brief, handedness, a complex human aspect that reflects the functional lateralization of the hemispheres, also interacts with the immune system. Given the role played by the brain in these processes, it is especially interesting to evaluate handedness and other body laterality preferences in patients with lesion to the central and peripheral nervous systems caused by immunological or other pathological factors and to clarify the properties of the EHI, the most widely used measure for handedness, in these patients. The specific objectives of this study were (a) to determine the relationships between hand, foot, and eye laterality preferences and clinical variables in patients with IM diseases and other neurological conditions (noIM) and (b) to test the properties of the EHI in these patients and healthy adults. We expected the IM disease patients to be more frequently left-handed or ambidextrous than the noIM disease patients and the EHI is an appropriate measure of body laterality preferences.

## 2. Materials and Methods

### 2.1. Participants

Adult patients with neurological disturbances associated with central or peripheral nervous system lesions or no detected lesions, either inpatient or outpatient, were selected. The exclusion criteria were primary psychiatric illnesses such as bipolar disorder, major depression and psychosis, systemic organ failure, and drug or alcohol abuse. Selected patients were assigned to three groups (IM diseases, noIM_brain, noIM_nobrain) based on the diagnosis of neuro-immunological disease or noIM disease affecting the brain or the spinal cord/peripheral nervous system. Healthy subjects, including hospital staff and patients’ relatives, constituted the control group; mild transitory symptoms such as tension headache and anxiety/depression prior to assessment were admitted. All participants gave their informed consent prior to clinical and instrumental assessment. This study was performed in accordance with the ethical standards laid down in the 1964 Declaration of Helsinki and its later amendments.

### 2.2. Assessment

Assessments were performed by a neurologist (ARG). Both the patients and healthy controls were asked questions regarding two aspects: (a) subjectively perceived hand preference (i.e., self-evaluated right- or left-handedness or ambidexterity in daily life) and (b) positive family history of left-handedness or ambidexterity. Patients’ clinical history also included stressful events preceding disease onset. The complete version of the EHI [66], comprising 20 items for the hands and 1 item for foot and eye activities, was administered to all participants, and we answered any questions patients had if they found certain items to be unclear. They were asked to consider their lateral preference for each activity. If the preference was so strong that they would never try to use the contralateral hand, foot, or eye, the examiner attributed a score equal to two (which corresponds to ++, given by the self-assessment), while a score equal to one was attributed to both hands, feet, or eyes if the action was performed at either sides. Ten item scores (writing, painting, throwing, using scissors, tooth brushing, using a knife without fork, using a spoon, holding the broom, lighting a match, opening a box) were considered to compute the handedness laterality quotient (LQ) based on the following formula: [(R − L)/(R + L)] × 100. The LQ is a percentage ranging from −100 to +100, allowing one to classify left-handedness (<−40), ambidexterity (−40 to +40), and right-handedness (>+40) [66].

### 2.3. Statistical Analysis

A one-way analysis of variance (ANOVA) was used to compare chronological age, while chi^2^ test assessed the distribution of females and males, participants with subjective left-handedness or ambidexterity, and participants with a family history for left-handedness and ambidexterity between the patient and control groups. Separate one-way ANOVAs compared age of disease onset, disease duration, and number of stressful events prior to illness between the patient groups.

A multivariate ANOVA with chronological age and sex as covariates (MANCOVA) compared the EHI scores for the 20 hand-related items between the patient and control groups; based on Bonferroni’s rule for 20 pairwise comparisons, the significance level was set at *p* < 0.002; an ANCOVA with the same covariates compared the LQ scores between the patient and control groups. Separate MANCOVAs compared the foot and eye item scores.

A multiple stepwise regression analysis was conducted to explore the power of chronological age, sex, and family history of left-handedness or ambidexterity to determine the EHI factors and LQ in the whole participants group. In the whole patients group, regression analysis evaluated the relationship of the LQ and EHI factors to diagnosis (IM, noIM_brain, noIM_nobrain), age of seizure onset, and disease duration.

A factor analysis based on a principal component analysis with an eigenvalue equal to one was used to evaluate the distribution of 22 EHI scores; each score was attributed to one factor based on a factor loading ≤ 0.5. Cronbach’s alpha test provided an index of internal consistency of all EHI scores, while chi^2^ tested the association between the LQ classes (right-handedness, left-handedness, ambidexterity) and the subjective perception of left-handedness or ambidexterity (yes/no).

## 3. Results

### 3.1. Participants

Three hundred thirty-four patients who had received compulsory schooling, with or without a high school degree, were evaluated and divided into three groups according to the diagnosis provided by the treating neurologist. The first group (IM) included patients with immune-mediated diseases of the central or peripheral nervous system (multiple sclerosis, recurrent optic neuritis, retrobulbar optic neuritis, systemic lupus erythematosus or other cerebral vasculitis, sequelae of post-infectious encephalitis, meningo-radiculo-neuritis, polyneuritis, myasthenia). The second group (noIM_brain) included patients with non-immune-mediated brain diseases (venous angioma, glioma, lymphoma, migraine, olivopontocerebellar atrophy, sarcoidosis, pseudobulbar syndrome, epilepsy, sequelae of head trauma, chronic ischemia, motor neuron disease, spastic paraparesis). The third group (noIM_nobrain) included patients with non-immunological diseases of the spinal cord or peripheral nervous system (tumor, chronic cervical or lumbar radiculopathy, diabetic polyneuritis, trigeminal neuralgia, VIII cranial nerve neuropathy, peripheral asymmetric or segmental neuropathy, cervical myelopathy).

Forty healthy subjects were the controls. Sex distribution differed among the four groups [chi^2^(3) = 9.52, *p* = 0.023] due to there being more women in the control and IM groups. There was also a significant difference in chronological age [F(3370) = 5.75, *p* < 0.001] due to the younger age of the IM disease patients compared with the noIM_brain patients (*p* < 0.001). On the contrary, there were no between-group differences in subjective perception of left-handedness or ambidexterity and number of participants with a family history of left-handedness or ambidexterity. Comparisons of three patients’ groups yielded significant differences for the age of disease onset [F(2331) = 13,29, *p* < 0.001], disease duration [F(2331) = 5.42, *p* = 0.005], and number of stressful events prior to illness [F(2331) = 6.21, *p* = 0.002] due to earlier disease onset, longer disease duration, and more pre-illness stressful events in the patients with IM diseases in comparison with the noIM disease patients (Table 1).

### 3.2. Psychometric Properties of the Edinburg Handedness Inventory

#### 3.2.1. Reliability and Convergent Validity

Cronbach’s test, used to analyze all EHI scores in all participants, yielded an alpha value of 0.77, indicating good internal consistency among the scores for the 20 hand-related items. chi^2^ showed a significant association between the number of participants classified by the LQ (left-handed, right-handed, ambidextrous) and by subjective perception of left-handedness or ambidexterity (yes/no) (chi^2^ = 147.38, *p* < 0.001).

#### 3.2.2. Factor Analysis

Table 2 shows the factors derived from 24 EHI scores for the hands, feet, and eyes in the group of all participants. These factors explained 71% of the variance, yielding five factors (Hand Transitive, Hand Refined, Hand Median, Foot, Eye). Hand Transitive collected gestures performed with one or both hands in peri-personal space (the space where objects can be reached by extending an arm); Hand Refined included writing, painting, and using a spoon, which are complex gestures of the distal part of an arm; and Hand Median included two bimanual gestures (using a broom and using a rake) applied to a long stick in peri-personal and extra-personal space (outside the length of an arm). The Foot and Eye factors included the scores for the right and left foot and eye.

#### 3.2.3. Between-Group Comparisons of Edinburgh Handedness Inventory Scores

**Figure 1:**
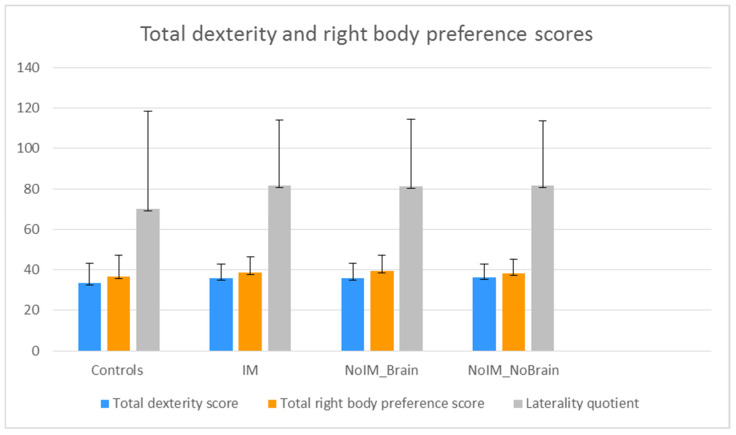
Total dexterity, total right body preference, and laterality quotient.

Our MANCOVA comparing the 20 hand items scores of the EHI between the patient and control groups yielded a global influence for chronological age (Pillai’s value = 0.08, F = 1.58, *p* = 0.05) and group (Pillai’s value = 2.40, F = 1.53, *p* = 0.007). Through carrying out post hoc ANOVAs, we found that chronological age showed significant effects on writing (*p* < 0.001) and painting (*p* = 0.003) and minor effects on throwing (*p* = 0.01), using a knife and fork (*p* = 0.009), and using a tennis racket (*p* = 0.01) or scissors (*p* = 0.02). Post hoc between-group comparisons revealed a significant difference for pencil use (*p* = 0.002) due to lower scores (i.e., greater use of left hand) in the IM group (1.63 ± 0.73) compared to the noIM_brain (1.90 ± 0.44) and noIM_nobrain (1.84 ± 1.49) groups. The LQ only showed a significant influence for chronological age (F(1) = 7.33, *p* = 0.007), with no effect for sex and group (Figure 1); our non-parametric Kruskal–Wallis one-way ANOVA confirmed the lack of between-group differences (chi^2^ = 1, *p* = 0.80). Likewise, the distribution of left-handed, ambidextrous, and right-handed participants was similar between the patient and control groups (chi^2^ = 6.92, *p* = 0.33), with there being more ambidextrous than left-handed patients in all groups.

Chronological age also affected the foot score (Pillai’s value = 0.021, F = 7.73, *p* = 0.006), whereas sex and group showed no effects, and no effect on the eye score was found for these variables.

Comparisons of the total dexterity (all right-hand activities) and total right body (hand, foot, and eye) scores confirmed a global influence for chronological age (Pillai’s value = 0.024, F = 3.05, *p* = 0.029) and no influence for sex or group (Figure 1). In line with these outputs, our MANCOVA comparing the EHI factors revealed a significant influence for chronological age (Pillai’s value = 0.04, F = 3.22, *p* = 0.007), which impacted on the Hand Refined (*p* = 0.002) and Foot factors (*p* = 0.038).

Our MANCOVA comparing the three patient groups with age of disease onset and disease duration as the covariates revealed a significant global influence for age of disease onset (Pillai trace = 0.04, F = 2.53, *p* = 0.03) and disease duration (Pillai trace = 0.04, F = 2.95, *p* = 0.01), which had significant effects on Hand Refined (*p* = 0.02) and Hand Transitive (*p* = 0.003), respectively.

#### 3.2.4. Predictors of the Edinburgh Handedness Inventory Factors and Laterality Quotient

In the whole participants group, our multiple stepwise linear regression analysis entering demographic variables and family history of left-handedness or ambidexterity showed that chronological age (B = 0.31, Beta = 0.14, *p* = 0.007) and family history (B = −7.13, Beta = −0.10, *p* = 0.05) cooperated to predict the LQ. Separate regression analyses of the EHI factors revealed that Hand Refined was predicted by chronological age (B = 0.10, Beta = 0.16, *p* = 0.002), Hand Transitive was predicted by sex (B = −0.21, Beta = −0.10, *p* = 0.04) and family history of left-handedness or ambidexterity (B = −0.21, Beta = −0.10, *p* = 0.05), and Foot was mildly affected by chronological age (B = 0.06, Beta = 0.10, *p* = 0.06), whereas no variables could predict the Hand Median and Eye factors.

In the patient groups, our multiple stepwise linear regression analysis entering clinical variables [age of disease onset, disease duration, family history of left-handedness or ambidexterity, diagnosis (IM, noIM_brain, noIM_nobrain)] revealed that the LQ was predicted by age of disease onset (B = 0.29, Beta = 0.15, *p* = 0.01), Hand Transitive by disease duration (B = −0.02, Beta = −0.17, *p* = 0.004) and Hand Refined by age of disease onset (B = 0.01, Beta = 0.14, *p* = 0.02). Hand Median (B = −0.23, Beta = −0.11, *p* = 0.04) and Foot (B = −0.21, Beta = −0.11, *p* = 0.05) were predicted by family history of left-handedness or ambidexterity, but no relationships were found between any clinical variables and the Eye factor. Membership to a diagnostic group did not impact on the EHI factors. Accordingly, our logistic regression analysis showed that the EHI factors and LQ could not predict membership to the patient and control groups.

## 4. Discussion

This clinical study provided three main findings: the EHI had satisfactory psychometric properties; lateralization of hand and foot activities correlated with chronological age and clinical variables; and handedness for complex activities distinguished neurological patients with IM diseases from those with noIM brain diseases.

In 374 Italian subjects, the EHI items showed good internal consistency, as an index of reliability, and the LQ classes (left-handed, right-handed, ambidextrous) were significantly associated with those identified by subjective perception of handedness, suggesting that the EHI also has convergent validity with daily activities. The items on the EHI demonstrated a clear division into five factors categorizing a wide range of movements: hand movements performed in peri-personal space (Hand Transitive), hand movements involving verbal and visual symbols and fine motor patterns (Hand Refined), bimanual movements in peri-personal and extra-personal middle space (Hand Median), foot movement (Foot), and eye fixation (Eye). This indicates good structural validity, in contrast to a one-solution factor for all items in the EHI [67,68]. Discrepancies between study results may reflect differences in the assessment of certain activities and in participants. For example, Espirito-Santo et al. [69] assessed hand preferences and the LQ in healthy subjects, whereas this study examined all items related to the hands, feet, and eyes and analyzed a range of laterality scores in patients with a neurological disease and healthy subjects.

Based on the LQ, 1% or 2% of neurological patients were left-handed, and 7% to 10% were ambidextrous, with an average percentage of non-right-handed subjects (4.8%) lower than that of healthy left-handed subjects reported in previous Italian studies, from 6.4% [85] to 7.9% [86], and in studies from other countries (10.6%) [1]. In contrast, 11% of the healthy subjects were non-right-handed (5% left-handed, 17% ambidextrous) a similar percentage to that of healthy non-Italian subjects [1]. The low frequency of left-handedness in patients with IM neurological diseases suggests, in particular, that left-handedness has no substantial relationship with the neural modulation of the immune system. The variability in the LQ in this study and those conducted in previous studies suggests that handedness may be influenced by country-related factors.

Chronological age proved to have significant effects on all hands and feet item scores and cumulative scores (i.e., total ambidexterity, total right body, and LQ) provided by the EHI. In addition, it showed a significant influence on the Refined Hand and Foot factors. This means that the lateral preference of hand and foot may change over the course of life, perhaps due to experience and repeated movements and changes in brain connectivity. In this regard, in the first year of life, handedness is not an established function, as children predominantly use their right hand, but they also experience recurrent periods of left-handedness or bilaterality [87,88,89], with stabilization between the third and seventh year [90]. Aging also affects hand function due to declines in motor strength, the ability to control and maintain precise hand and finger postures, manual speed, and tactile and proprioceptive sensations [91]. Educational, sociocultural, and occupational factors can influence handedness in adult life [37,87]. Hence, manual skills, hand use, and foot preference undergo updating processes under the influence of use, culture, aging, and environment, which helps us understand why handedness is an indirect indicator of hemispheric functional lateralization. Of note, chronological age showed a significant influence on the Refined Hand factor, whose main loadings were represented by writing and painting, and writing and painting also were the single items most significantly influenced by chronological age in the whole participant population. These results highlight a novel aspect: hand activities involving abstraction and imagination, as well as verbal and visual symbols, are mostly correlated with chronological age, perhaps as a function of their long-term reinforcement and retention in semantic and implicit memory stores. In fact, chronological age has shown minor or insignificant effects on activities involving one or more objects, such as a fork, tennis racket, and scissors. Handedness for refined symbolic actions may thus be strengthened throughout life, while lateralization for transitive activities, which are object-driven, could be more stable. Notably, in the entire population of participants, individual foot items and Foot factor scores were also significantly affected by chronological age. All this suggests that the lateralization of hand and foot activities is not a fixed human aspect, but that it can change over the course of life as a consequence of learning and practice, especially for abstract and symbolic activities.

The patients with IM diseases scored lower than the noIM disease patients in pencil use, indicating a tendency for left-handedness. Typical pencil use often requires the use of an eraser, as pencils are commonly used for writing or drawing tasks that can be modified, highlighting their temporary nature and adaptability. In addition, the pencil is used only by the hands, so it is argued that pencil use is a specific index of handedness, and the low score in the IM disease patients may support the association between left-handedness and immunological changes in the nervous system. However, given the low frequency of non-right-handed patients in the IM group, this result alone does not seem sufficient for making inferences about the asymmetric neural modulation of immune responses or Geschwind and Behan’s theory [50]. The present study’s results are not consistent with the high incidence of left-handedness among individuals with immune disorders [54] or with the mirror changes in cellular immune responses after epilepsy surgery in the dominant and nondominant hemisphere [82] and in immune regulators after left hemisphere (depression) and right hemisphere (potentiation) damage [43,44,45,48].

Notably, in the whole group of patients, the factors Hand Refined and Hand Transitive were significantly correlated with age of disease onset and duration of disease, implying that early disease onset and a long disease duration may induce changes in handedness. The impact of chronic diseases of the nervous system on handedness may depend on various factors, such as brain plasticity and body compensation. The time window of neurodevelopment coincides with the age of onset of many neurological conditions, such as epilepsy, inflammatory diseases, dysplasia, and early-onset brain tumors, thus creating a complex interaction between brain plasticity triggered by physiological neurodevelopment and pathology [92]. Most importantly, the influence of chronic neurological disease, together with chronological age, may overcome the primary relationships between the neural modulation of immune responses and handedness. It appears that the “time” factor helps to explain the discrepancies between the findings on left-handedness in this study and previous studies on non-neurological patients and healthy subjects [50,54].

Of note, compared with other patient groups, the IM group included more females than males, consistent with the larger number of females in populations with IM diseases [56] and the impact of female sex hormones on the pathogenesis of autoimmune diseases [57]. The IM disease patients were also younger than the noIM_brain patients and had an earlier disease onset and longer disease duration than the patients without IM diseases. In addition, the IM group reported more stressful events than the patients without IM diseases, which replicates findings on the effects of stress on immunity [33,34]. It thus appears that female sex, young age, early disease onset, and prior stressful events may negatively affect the prognosis of IM neurological diseases.

The results of this study must be interpreted while taking into account this study’s limitations. The number of healthy subjects was low compared with the patient groups, and although covariates were included in the between-group comparisons and the significance level was set according to the number of comparisons, the control group is partially representative of the general healthy population. Since this was primarily a clinical study, we did not analyze the results of instrumental tests such as neuroimaging and electroencephalograms, although they were used to obtain diagnoses.

## 5. Conclusions

Handedness is not a fixed human aspect. In patients with neurological conditions, it may be determined not only by genetics, personal experience, and sociocultural standards but also by chronic diseases of the central and peripheral nervous system and age over the course of life. This may explain why, unlike systemic immunological diseases, IM neurological diseases are not closely associated with left-handedness. The EHI is a reliable and valid measure of the lateralization of hand, foot, and eye activities to contextualize the neurological picture; therefore, these results extend the Italian normative data sets. Further studies are needed to elucidate changes in handedness throughout life and its relationship with neurological diseases.

## Figures and Tables

**Table 1:** Demographic and clinical aspects.

	IM Diseases (*n* = 135)	noIM Brain Lesions (*n* = 143)	noIM Spinal Cord, Root, or Nerve Lesions (*n* = 56)	Healthy Subjects (*n* = 40)
Females	83 (61%)	64 (48%)	25 (48%)	29 (72%)
Chronological age	39.77 ± 14.28	46.97 ± 16.45	45.95 ± 15.33	41.95 ± 13.98
Participants with familial left-handedness or ambidexterity	54 (40%)	54 (38%)	16 (29%)	16 (40%)
Participants with subjective left- handedness or ambidexterity	23 (17%)	27 (19%)	9 (16%)	12 (30%)
Laterality quotient	81.84 + 32.44	81.26 + 33.16	81.43 + 32.05	69.50 + 48.25
Left-handed	3 (2%)	2 (1%)	1 (2%)	2 (5%)
Ambidextrous	9 (7%)	14 (10%)	4 (7%)	7 (17%)
Right-handed	123 (91%)	127 (89%)	51 (91%)	31 (78%)
Stressful events	0.16 ± 0.58	0.01 ± 0.12	0.02 ± 0.13	-
Age of disease onset	32.04 ± 14.19	41.94 ± 18.70	40.66 ± 16.69	-
Disease duration (years)	7.81 ± 8.82	4.75 ± 7.26	5.29 ± 7.38	-

**Table 2:** Factors yielded by all Edinburgh Handedness Inventory scores in neurological patients and healthy subjects.

	Hand Transitive	Hand Refined	Hand Median	Foot	Eye
Throwing	0.58				
Using scissors	0.68				
Combing	0.71				
Tooth brushing	0.60				
Beating with the hammer	0.73				
Screwdriver	0.71				
Tennis racket	0.70				
Lighting a match	0.68				
Opening a box (cover)	0.68				
Distributing the cards	0.67				
Threading a needle	0.73				
Using a knife without a fork	0.66				
Using a knife and fork	0.55				
Using a pencil	0.70				
Wrapping a thread	0.70				
Writing		0.83			
Painting		0.83			
Using a spoon		0.59			
Holding the broom			0.91		
Holding the rake			0.90		
Right kick				0.91	
Left kick				−0.91	
Right eye					0.97
Left eye					−0.97

## Data Availability

The data that support the findings of this study are available from the corresponding author due to privacy reasons.
